# Supplementary material for: The Survey Measure of Psychological Safety and Its Association with Mental Health and Job Performance: A Validation Study and Cross-Sectional Analysis
Source: Int J Environ Res Public Health. 2022 Aug 11;19(16):9879. doi: 10.3390/ijerph19169879 (PMC9407795; doi:10.3390/ijerph19169879)
Supplement: Supplementary file 1 [file ijerph-19-09879-s001.zip › ijerph-1813602-supplementary.pdf]

**Supplementary Materials: The final version of the Japanese Psychological Safety Scale.**

問：以下の質問に、「1=全くそう思わない」から「7=非常にそう思う」の評価で答えてください。

セクション 1. あなたの**チームのリーダー（またはチームをまとめる上司）**について、以下の質問にお答えください。

Please respond to the following questions by indicating your response between 1 = strongly disagree and 7 = strongly agree.

Section 1. Please answer the following questions in relation to your **team leader.**

| | 1 全くそう思わない strongly disagree | 2 | 3 | 4 | 5 | 6 | 7 非常にそう思う strongly agree |
|---|---|---|---|---|---|---|---|
| １．私は、仕事での自分の役割についての疑問やわからないことがあった場合、チームのリーダー（または上司）に聞くことができる<br>1 If I had a question or was unsure of something in relation to my role at work, I could ask my team leader. | | | | | | | |
| ２．私は、仕事上の問題について自分の意見をチームのリーダー（または上司）に伝えることができる<br>2 I can communicate my opinions about work issues with my team leader. | | | | | | | |
| ３．私は、個人的な問題や反対意見をチームのリーダー（または上司）に伝えることができる<br>3 I can speak up about personal problems or disagreements to my team leader. | | | | | | | |
| ４．私は、新しいプロジェクトや手順の変更について、チームのリーダー（または上司）に提案したりアイディアを伝えたりすることができる<br>4 I can speak up with recommendations/ideas | | | | | | | |

| | | | | | | | |
|---|---|---|---|---|---|---|---|
| for new projects or changes in procedures to my team leader. | | | | | | | |
| ５．私は、このチームで失敗をしてしまったとしても、チームのリーダー（または上司）に安心して伝えることができる<br>5 If I made a mistake on this team, I would feel safe speaking up to my team leader. | | | | | | | |
| ６．私は、同僚が誤ったことをしているのを見た場合、チームのリーダー（または上司）に安心してそのことを伝えることができる<br>6 If I saw a colleague making a mistake, I would feel safe speaking up to my team leader | | | | | | | |
| ７．私が自分の意見を言うと、チームのリーダー（または上司）はその意見を尊重して聞いてくれる<br>7 If I speak up/voice my opinion, I know that my input is valued by my team leader. | | | | | | | |
| ８．チームのリーダー（または上司）は、私が新しい仕事を引き受けたり、経験のない仕事のやり方を学んだりすることを奨励し、サポートしてくれる<br>8 My team leader encourages and supports me to take on new tasks or to learn how to do things I have never done before. | | | | | | | |
| ９．私が自分の所属する組織（会社など）で問題を抱えた場合、チームのリーダー（または上司）が私の味方になってくれると信じている<br>9 If I had a problem in this company, I could depend on my team leader to be my advocate. | | | | | | | |

セクション 2. あなたの**同僚/チームの他のメンバー**について、以下の質問に答えてください。

Section 2. Please answer the following questions in relation to your **peers/the other members of your team**

| | 1 全くそう思わない strongly disagree | 2 | 3 | 4 | 5 | 6 | 7 非常にそう思う strongly agree |
|---|---|---|---|---|---|---|---|
| １０．私は、仕事での自分の役割についての疑問やわからないことがあった場合、同僚に聞くことができる<br>10 If I had a question or was unsure of something in relation to my role at work, I could ask my peers. | | | | | | | |
| １１．私は、仕事上の問題について自分の意見を同僚に伝えることができる<br>11 I can communicate my opinions about work issues with my peers. | | | | | | | |
| １２．私は、個人的な問題を同僚に伝えることができる<br>12 I can speak up about personal issues to my peers. | | | | | | | |
| １３．私は、新しいプロジェクトや手順の変更について、同僚に提案したりアイディアを伝えたりすることができる<br>13 I can speak up with recommendations/ideas for new projects or changes in procedures to my peers. | | | | | | | |
| １４．私は、このチームで失敗をしてしまったとしても、同僚に安心して伝えることができる<br>14 If I made a mistake on this team, I would feel safe speaking up to my peers. | | | | | | | |
| １５．私は、同僚が誤ったことをしているのを見た場合、同僚に安心してそのことを伝えることができる<br>15 If I saw a colleague making a mistake, I would feel safe speaking up to this colleague. | | | | | | | |
| １６．私が自分の意見を言うと、同僚はそ | | | | | | | |

| | | | | | | | |
|---|---|---|---|---|---|---|---|
| の意見を尊重して聞いてくれる<br>16 If I speak up/voice my opinion, I know that my input is valued by my peers. | | | | | | | |

セクション 3. あなたの**チーム全体**について、以下の質問にお答えください。

Section 3. Please answer in relation to your **team as a whole**

| | 1 全くそう思わない strongly disagree | 2 | 3 | 4 | 5 | 6 | 7 非常にそう思う strongly agree |
|---|---|---|---|---|---|---|---|
| １７．このチームのメンバーには、助けてほしいとお願いしやすい<br>17 It is easy to ask other members of this team for help. | | | | | | | |
| １８．チーム内の仕事上の問題について、お互いに情報を交換しあえる<br>18 People keep each other informed about work-related issues in the team. | | | | | | | |
| １９．チーム全体で情報を共有するための取り組みがされている<br>19 There are real attempts to share information throughout the team. | | | | | | | |

*Note:* The original (English) version of the scale was cited from O'Donovan, R.; Van Dun, D.; McAuliffe, E. Measuring psychological safety in healthcare teams: developing an observational measure to complement survey methods. *BMC Med. Res. Methodol.* **2020**, *20*, 203.
